# Acceptability of the COVID-19 Vaccine among Adults in Saudi Arabia: A Cross-Sectional Study of the General Population in the Southern Region of Saudi Arabia

**DOI:** 10.3390/vaccines10010041

**Published:** 2021-12-29

**Authors:** Yahya S. Alqahtani

**Affiliations:** Department of Pharmaceutical Chemistry, College of Pharmacy, Najran University, Najran 66462, Saudi Arabia; ysalmuneef@nu.edu.sa; Tel.: +966-500980592

**Keywords:** COVID-19 vaccine, vaccine acceptance, vaccine hesitancy, immunisation, Saudi Arabia

## Abstract

Vaccines afford protection against infectious diseases. However, a sizeable part of the population refuse vaccinations and continue to dispute the evidence supporting vaccinations. The objective of this study was to determine the prevalence of COVID-19 vaccination uptake and its determinants among the Saudi population in the southern region of Saudi Arabia. A cross-sectional survey studied COVID-19 vaccine acceptance in adults in Saudi Arabia, targeting the general population in the southwestern region. Data were collected through an online survey questionnaire tool. All data were analysed using SPSS version 23.0. The majority (57.29%) of the participants were willing to receive the new COVID-19 vaccine, whereas almost 64% believed it is necessary to take the COVID-19 vaccine to protect oneself and that the vaccine is safe, efficient and effective. The data showed that perceived risk of COVID-19 (*p* = 0.015), history of previous vaccination against seasonal influenza (*p* = 0.000), and trust in the healthcare system (*p* = 0.025) were significant predictors for COVID-19 vaccine acceptance. We conclude that participants’ trust in the healthcare system, perceived risk of contracting COVID-19, and history of previous vaccination against seasonal influenza were significant predictors for COVID-19 vaccine acceptance. Knowing the acceptance rates for the COVID-19 vaccination can aid state agencies, medical practitioners, and other entities in reducing the impact of vaccine avoidance.

## 1. Introduction

The proven success of vaccines in preventing disease, disability, and death from vaccine-preventable ailments has rendered vaccines the focus of attention in the fight against new emerging infections and illnesses. Vaccines now afford protection even against several cancer types. Indeed, an ample array of proof advocates immunisation and vaccination [1]. However, people in several parts of the world refuse vaccinations and continue to dispute the evidence supporting vaccinations; Saudi Arabia is no exception [2,3]. Vaccine hesitancy (VH) is defined as people’s unwillingness to receive vaccines or a delay in vaccine acceptance, despite vaccination drives offered by health administrations. This attitudinal aspect is context- and vaccine-specific and emerges from a multifaceted judgment-making procedure influenced by many factors, which can be divided into three categories: convenience, complacency, and confidence [4].

Health literacy is a broad notion that refers to people’s abilities to fulfil the complicated demands of modern health [5]. “Health literacy is linked to literacy and entails people’s knowledge, motivation, and competence to access, understand, appraise, and apply health information to make judgments and take decisions in everyday life concerning healthcare, disease prevention, and health promotion to maintain or improve quality of life throughout the life course,” according to the Sørensen Integrated Model [6].

The severe acute respiratory syndrome coronavirus 2 (SARS-CoV-2) pandemic, universally designated “COVID-19”, had infected more than 105 million people in 144 countries as of 4 February 2021 [5,7,8]. COVID-19 poses a substantial threat to the healthcare system, along with devastating economic consequences across the globe, to which the Kingdom of Saudi Arabia (KSA) is not immune [9,10]. Pandemics have struck Saudi Arabia in the past, notably the Middle East respiratory syndrome coronavirus (MERS-CoV). The continuing COVID-19 epidemic [11,12] has quickly spread across the Kingdom, resulting in 545,992 laboratory-confirmed cases and 8623 deaths as of 12 September 2021.

Developing a vaccine is considered the most critical intervention to fight such pandemics. Bringing a new vaccine to the market is a long process that involves initial product development, pharmaceutical quality determination, and pre-clinical evaluation in laboratory and animal models, followed by three phases of clinical trials, which involve clinical efficacy and safety studies. At this stage, the data submitted by the developers are evaluated, and if found satisfactory, the product is authorized by regulatory agencies to be used in the public at a global level, subject to fulfilment of other requirements. However, to control the pandemic, the development of COVID-19 vaccines has been carried out rapidly utilizing the knowledge gained with existing vaccines. Though the development of COVID-19 vaccines was fast-tracked, all steps were taken to ensure their safety and effectiveness. Furthermore, even after the vaccine is released into the market, various regulatory agencies worldwide continuously monitor its ongoing safety and use in real life [13,14].

Nevertheless, information related to the public’s attitude and perception towards COVID-19 vaccine acceptability is limited, especially in the Middle Eastern countries. Studies report that several factors influence the acceptance of a new vaccine [15,16,17,18,19]. These include the efficacy and safety of the vaccine, unfavourable health outcomes, misconceptions and misinformation about the need for vaccination, trust deficit in the healthcare system, and absence of knowledge regarding vaccine-preventable diseases amongst the community [15,16]. Misinformation steering towards vaccine hesitancy could put public health in jeopardy and cause hurdles in responding to the current crisis. Thus, the need of the hour is to assess the public’s perception regarding vaccine acceptance and to frame specific strategic interventions to create a positive public perception towards the COVID-19 vaccine.

The Ministry of Health in Saudi Arabia has taken concrete steps to ensure that the COVID-19 vaccination drive is successful and the most vulnerable people receive the vaccine on a priority basis. So far, the public has shown a strong willingness to receive the COVID-19 vaccination, with more than 39 million people receiving the vaccine [20]. In addition, to encourage vaccination, the Ministry of Commerce has urged retailers such as electronic and retail stores, cafés, and restaurants to participate in the effort by offering discounts to consumers who have received the coronavirus vaccination.

The findings from this research will provide data central to the development of evidence-based interventions to improve health literacy further and reduce vaccine hesitancy in the general public in Saudi Arabia. The data may be useful for policymakers for the effective implementation of the public vaccination drive during the COVID-19 pandemic.

The current study’s specific objectives were to find correlations of COVID-19 vaccination acceptance, as evaluated by individuals’ desire to obtain the vaccine (i.e., vaccine acceptability). The factors that would influence the participants’ decisions about receiving a COVID-19 vaccine were examined. The willingness of the target population to volunteer for a COVID-19 vaccine clinical trial in Saudi Arabia was assessed.

## 2. Methodology

### 2.1. Study Design

A cross-sectional survey studied COVID-19 vaccine acceptance in adults in Saudi Arabia, targeting the general population in the southwestern region.

### 2.2. Eligibility Criteria

Eligibility requirements included being at least 18 years old and currently residing in Saudi Arabia. Participants were recruited for this study when they were determined to be eligible and had given their informed consent before completing their online study survey.

### 2.3. Data Collection

Data were collected through an online survey questionnaire tool. The tool link was distributed to the participants through online social media platforms such as WhatsApp, Twitter, and email. A simple random and snowball sampling technique was used to recruit the study participants.

### 2.4. Study Tool

We developed survey items on vaccination acceptance based on past research involving vaccination behaviours [19,21,22,23,24,25]. The participants were given a questionnaire that was divided into three parts: the first part collected demographic and background information; the second part focused on participants’ knowledge and perceptions of COVID-19, willingness to accept the COVID-19 vaccine, trust in the health system, and willingness to volunteer for a COVID-19 vaccine clinical trial in Saudi Arabia; and the third part included three questions to assess vaccine hesitancy.

### 2.5. Reliability Analysis

Pilot research was conducted among the study population to assess the study questionnaire’s reliability and validity. A total of 30 respondents were recruited (10 from each region). The questionnaire’s reliability test resulted in a Cronbach’s alpha of 0.871, suggesting that it has extremely excellent internal consistency. The pilot research participants were included in the final data analysis.

### 2.6. Sample Size Calculation

The three major cities (Najran, Abha/Khamis Mushait, and Jizan) in the southwestern region of Saudi Arabia included in this study have a combined population of approximately 962,210. With a predefined margin of error of 5% and a confidence level of 95%, the sample size was estimated using an online sample size calculator (http://www.raosoft.com/samplesize.html (accessed on 11 February 2021). The sample size was calculated to be 384.

### 2.7. Ethical Issues

By clicking the questionnaire link, the participants were auto-directed to the informed consent page. The study objectives were explained to participants on the consent form page, which was followed by the survey questionnaires. The participants’ rights and integrity were respected throughout this investigation. The study was authorised by the Scientific Research Ethics Committee at Najran University, with the number 442-41-34168-DS. The participants were informed that their participation in the study was entirely voluntary and that they may leave at any time. The Helsinki Declaration’s principles were followed in this investigation.

### 2.8. Statistical Analysis

Descriptive statistics (frequencies, means) were calculated for all variables. Correlations of COVID-19 vaccine acceptability were identified by using relative risk regression models. We used ordinal regression models to look at demographic and attitude variables that predicted respondents’ desire to obtain COVID-19 vaccines. All data were analysed using SPSS version 23.0.

## 3. Results

### 3.1. Demographic Characteristics of the Respondents (n = 391)

The majority (48.34%) of the study participants were young, between 18 and 29 years of age. More than two-thirds were male, and 96% were Saudi citizens. Two-thirds of the participants who resided in rural areas were graduates. One-third came from medical professional backgrounds. More than 50% of the study participants had undergone a COVID-19 test at least once; however, only 12.79% had been previously diagnosed with COVID-19. Newsletters and SMS circulated by the Ministry of Health, KSA were the major source of information regarding the COVID-19 vaccine for most (58.1%) of the participants. Table 1 presents the demographic characteristics of the respondents.

### 3.2. Health Related Characteristics Vaccine Acceptability of the Respondents:

Table 2 presents the health-related characteristics of the respondents. The majority (88.24%) of the participants did not suffer from any chronic disease, and less than half had received an influenza vaccine during the current influenza season or intended to receive the influenza vaccine in the following season. Around 30% said they would receive the seasonal influenza vaccine in the context of the COVID-19 pandemic. More than half were afraid of COVID-19 and felt at risk of being infected by the new coronavirus. The majority (57.29%) of the participants were willing to receive the new COVID-19 vaccine, whereas almost 64% believed it was necessary to take the COVID-19 vaccine to protect oneself and that the vaccine was safe, efficient and effective. When asked about volunteering for a clinical trial associated with a new COVID-19 vaccine in Saudi Arabia, the majority (71.87%) did not agree to participate. More than 90% of the participants had full faith and trust in the Ministry of Health, Saudi Arabia, to make good decisions about reducing the spread of COVID-19. Less than 25% of the participants were vaccine hesitant. The term vaccine hesitancy covers delaying vaccines, outright refusals to vaccinate, using certain vaccines but refusing others, or accepting vaccines but remaining uncertain about their use.

### 3.3. Correlation Analysis Result

Five relationships were observed through Spearman’s Rho correlation analysis. The results showed no significant correlation between chronic disease and vaccine acceptability because the significant *p*-value (*p* = 0.966) is higher than 0.05, which accepts the null hypothesis (Table 3). Similarly, there is no significant correlation between vaccine hesitancy and vaccine acceptability (*p* = 0.991). However, there is a statistically significant correlation between trust in the health system and vaccine acceptability because the significant *p*-value (*p* = 0.008) is less than 0.05, which rejects the null hypothesis. Similarly, there is a statistically significant correlation between a history of previous vaccination against seasonal influenza and vaccine acceptability (*p* = 0.000). Furthermore, a statistically significant correlation was found between perceived fear or risk and vaccine acceptability (*p* = 0.008) (Table 3).

### 3.4. t-Test Results of Group Differences on Vaccine Acceptability

Four *t*-tests were conducted to observe the group differences of gender, nationality, profession, and degree of carefulness. However, no significant association was noted among these groups regarding vaccine acceptance. The results revealed no significant difference between males and females regarding vaccine acceptability as the *t*-value (0.501) is less than 1.96 and the *p*-value (*p* = 0.617) is higher than 0.05. Similarly, there is no significant difference between Saudi and non-Saudi nationals regarding vaccine acceptability (*t* = 0.172 and *p* = 0.863). Furthermore, there is no significant difference between medical and non-medical professionals regarding vaccine acceptability (*t* = 0.061 and *p* = 0.951). Likewise, there is no significant difference between being careful and careless about health regarding vaccine acceptability (*t* = 0.934 and *p* = 0.351). The detailed results are provided as Supplementary Files (Tables S1–S4).

### 3.5. ANOVA Test Results

ANOVA tests were conducted to observe any significant differences of various groups related to area and monthly income. Table 4 presents the results regarding vaccine acceptability based on three different areas of the southwestern region of Saudi Arabia: Najran, Abha/Khamis Mushait, and Jizan. No significant differences were observed between the three different areas concerning vaccine acceptability. Similarly, monthly income groups—less than SAR 5000, SAR 5001 to SAR 10,000, and above 10,000—revealed no significant differences among the three different monthly income groups concerning vaccine acceptability.

### 3.6. Regression Analysis Results

Table 5 presents the multiple linear regression results of four predictors of vaccine acceptability: vaccine hesitancy, trust in the health system, history of previous vaccination against seasonal influenza, and perceived fear or risk. From the path coefficient results, no statistically significant effect of vaccine hesitancy on vaccine acceptability was revealed. The *p*-value (0.136) is higher than 0.05 and the *t*-value (*t* = 1.493) is less than 1.96, which confirms no significant effect. The results showed a statistically significant effect of perceived fear or risk on vaccine acceptability (*t* = 2.609 and *p* = 0.015). There was a statistically significant effect of trust in the health system on vaccine acceptability. The *p*-value (0.030) is less than 0.05 and the *t*-value (*t* = 2.255) is higher than 1.96, which confirms a significant effect. Similarly, there was a statistically significant effect of a history of previous vaccination against seasonal influenza on vaccine acceptability (*p* = 0.000 and *t* = 11.890). The R-square results for vaccine acceptability revealed R2 = 0.547, or 54%, which confirms the adequate coefficient of determination.

## 4. Discussion

After the clinical development and availability of the COVID-19 vaccine, it now faces the challenge of public acceptance. The World Health Organization (WHO) has identified ten threats for global health, which include vaccine hesitancy and the risk of a pandemic [26]. In the current COVID-19 scenario, humanity is facing both threats. Vaccines are known to be highly cost-effective, successful public health tools for the prevention of pandemics that benefit public health by saving many lives. However, an effective vaccine alone will not stamp out the novel coronavirus, until vaccination programmes are implemented efficiently. The implementation of the vaccine programmes will be a decisive factor in overcoming the COVID-19 pandemic.

Vaccine hesitancy has been previously linked with other vaccines and has resurged [27,28], leading to the recent U.S. outbreaks of measles, which, until recently, had been largely wiped out there [29].

Another reason for vaccine hesitancy is the anti-vaccination movements taking place in different parts of the world, including America, Europe, Australia and New Zealand, to name a few. These movements usually mobilize events against vaccine acceptance and spread false and misleading information, claims and conspiracies. The curbing of these movements should be a priority set high to immunize the larger share of the population successfully [30,31,32,33].

Contrary to anti-vaccination movements’ baseless and unscientific propaganda, COVID-19 vaccines have shown significant benefits and outcomes in people inoculated against COVID-19. In an observational study from Israel, two doses of the Pfizer-BioNTech mRNA BNT162b2 vaccine successfully prevented symptomatic and asymptomatic SARS-CoV-2 infections across all age categories. Noted also was a reduction in COVID-19-related hospitalizations, disease severity and death [34]. Another study from England that involved adults (n = 156,930) aged 70 years and above vaccinated with either one dose of the Pfizer-BioNTech or Oxford-AstraZeneca ChAdOx1-S vaccine showed a significant reduction in symptomatic COVID-19 with further protection against severe disease. In addition, participants who had received one dose of BNT162b2 or ChAdOx1-S had a further reduced risk of hospitalization; reduced risk of death was observed in participants who had received one dose of BNT162b2 [35]. Recent research has also established the enhanced effectiveness of heterologous vaccination using ChAdOx1-S as a first dose followed by a boost with either BNT162b2 or Moderna mRNA-1273 as the second dose against symptomatic COVID-19 infection, including the delta variant [36].

In this research, we investigate the challenge posed by vaccine hesitancy to an available vaccine programme. What if the majority of the population decline the vaccine? What are the factors influencing vaccine acceptance? This study, the first of its kind in the southwestern region of Saudi Arabia, utilized an online self-administered questionnaire to collect the responses of the respondents, which included three major cities in the region (Najran, Abha/Khamis Mushait, and Jizan).

Our findings revealed that the majority of participants (57.29%) were willing to receive the vaccine; however, 24.55% were identified as vaccine hesitant. These findings are in line with other studies carried out in central Saudi Arabia [21], the U.S. [37], and China [38], which, however, reported a relatively higher percentage of vaccine acceptance compared to our findings. The studies conducted in China and the U.S. reported 72.5% and 80% VA, respectively. Similarly, the study conducted in Saudi Arabia reported 64.7% VA in the general population [21]. These findings suggest that the general Saudi population are receptive to the COVID-19 vaccine.

This study indicated that keenness to accept the COVID-19 vaccine is relatively high among middle-aged groups (30–49 years); however, it was not statistically significant. These findings are consistent with another study conducted in Saudi Arabia [21]. In our study, Spearman’s rho correlation analysis revealed that perceived fear or risk of being infected with COVID-19 was significantly (*p* < 0.01) associated with vaccine acceptance. These findings are in line with previously reported data in Indonesia [39] and Saudi Arabia [21]. Similarly, trust in the healthcare system was significantly (*p* < 0.05) associated with vaccine acceptance, which is consistent with previous studies conducted in Saudi Arabia [21]. Furthermore, a history of previous vaccination against seasonal influenza was significantly (*p* < 0.01) associated with COVID-19 vaccine acceptance. These findings corroborate a previous study conducted on healthcare workers (HCWs) in France [40] and Hong Kong [41]. Our study revealed that the intent to receive a COVID-19 vaccine exceeded the influenza vaccine rates in the current influenza season and the following season. This may be explained in part due to variation in perceived risk of contracting SARS-CoV-2, or seasonal influenza, as the general perception (fear or risk) of contracting influenza is relatively low compared to SARS-CoV-2 [42]. No significant differences were observed between medical and non-medical professions concerning vaccine acceptance. This could become a cause of concern in healthcare settings as HCWs have more and longer contact with COVID-19 patients than non-HCWs. These findings contrast with the previous studies conducted in France and Indonesia. The French study reported a relatively high rate of COVID-19 vaccine acceptance in HCWs compared to their counterparts [40]. Similarly, the Indonesian study reported that being a healthcare worker was associated with a higher COVID-19 vaccine acceptance rate [39]. Furthermore, our study reported no significant group differences between Saudi and non-Saudi, male and female, and careful and careless about health. In addition, no significant differences were observed between different groups of monthly income and areas, namely Najran, Abha/Khamis Mushait, and Jizan, concerning vaccine acceptance.

We performed regression analysis to determine how one variable affects another variable in our study. The data showed that perceived risk of COVID-19, history of previous vaccination against seasonal influenza, and trust in the healthcare system were significant predictors for COVID-19 vaccine acceptance. These findings agree with previous studies carried out in Saudi Arabia and elsewhere. According to these studies, the use of preventative health services such as vaccination has been linked to a higher level of trust in the healthcare system [21,22,43,44]. Thus, our findings are in line with earlier research that found links between government trust and vaccine uptake [45].

In Saudi Arabia, state-of-the-art healthcare facilities are provided by the Ministry of Health. The Ministry is active in public awareness efforts through newsletters and SMS, aimed at increasing public confidence in the vaccine’s efficacy and safety. The Ministry of Health provides free vaccination to all its citizens and resident population. In addition, our data revealed that vaccine hesitancy was not significantly associated with COVID-19 vaccine acceptability.

As of 10 September 2021, Saudi Arabia has administered 38.7 million doses of the COVID-19 vaccine, accounting for 16.1 million (47.1%) fully vaccinated individuals. The vaccination rate in Saudi Arabia is one of the highest in the world and has been commended by the WHO [46].

Our findings provide policymakers with information that may be used to design policies and plan for further enhancing of the vaccine acceptance rate.

Our study has a few limitations worth mentioning. Firstly, being a cross-sectional study, it represents the community’s response at a particular point in time. Vaccine acceptance rates may vary as the public perception regarding the vaccine changes in the future. Secondly, the study was carried out in the southern region of Saudi Arabia involving a sample size of 391, which might not be generalisable to the whole of Saudi Arabia. Thirdly, the study tool was distributed through web-based platforms such as email and WhatsApp, leading to the exclusion of people with lower educational and socioeconomic backgrounds. Finally, possible selection bias cannot be ruled out because of the self-selection recruitment process involved in online surveys.

## 5. Conclusions

Our findings conclude that the majority of the study participants were willing to receive the available COVID-19 vaccines in Saudi Arabia. Participants’ trust in the healthcare system, perceived risk of contracting COVID-19, and history of previous vaccination against seasonal influenza were significant predictors for COVID-19 vaccine acceptance. Communities’ perception of the dangers of COVID-19 should be increased through educational interventions by the health authorities concerned. Knowing the acceptance rates for the COVID-19 vaccination can aid state agencies, medical practitioners, and other entities in reducing the impact of vaccine avoidance.

## Figures and Tables

**Table 1:** Demographic characteristics of participants and vaccine acceptability (VA) (*n* = 391).

Demographic Characteristics	Frequency *n* (%)	VA *n* (%)
Age		
18 to 29 years	189 (48.34)	96 (50.79)
30–49 years	183 (46.80)	117 (63.93)
50–65 years	16 (4.09)	10 (62.5)
Above 65 years	3 (0.77)	1 (33.33)
Gender		
Male	270 (69.05)	161 (59.62)
Female	121 (30.95)	63 (52.06)
Nationality		
Saudi	375 (95.91)	215 (57.33)
Non-Saudi	16 (4.09)	9 (56.25)
City of residence		
Najran	148 (37.85)	82 (55.4)
Abha/Khamis Mushait	175 (44.76)	99 (56.57)
Jizan	68 (17.39)	43 (63.23)
Area of residence		
Rural	127 (32.48)	69 (54.33)
Urban	264 (67.52)	155 (58.71)
Education level		
High school	47 (12.02)	25 (53.19)
Diploma	40 (10.23)	29 (72.5)
Graduate	250 (63.94)	133 (53.2)
Postgraduate/Ph.D.	54 (13.81)	37 (68.51)
Monthly household income (SAR)		
Less than 5000	84 (21.48)	41 (48.8)
5000 to 10,000	127 (32.48)	69 (54.33)
More than 10,000	180 (46.04)	114 (63.33)
Profession		
Medical	141 (36.06)	85 (60)
Non-medical	250 (63.94)	139 (55.6)
Job sector		
Government	211 (53.96)	129 (61.13)
Private	37 (9.46)	25 (67.56)
Self-employed	12 (3.07)	5 (41.66)
Unemployed	131 (33.5)	62 (47.32)
Marital status		
Married	222 (56.78)	136 (61.26)
Unmarried/single	165 (42.2)	87 (52.72)
Separated/divorced/widowed	4 (1.02)	1 (25)
Smoking status		
Smoker	48 (12.28)	36 (75)
Non-smoker	320 (81.84)	175 (54.68)
Ex-smoker	23 (5.88)	13 (56.32)
Health status		
Excellent	285 (72.89)	168 (58.94)
Good	90 (23.02)	48 (53.33)
Fair	16 (4.09)	8 (50)
Degree of carefulness about health		
Careful	344 (87.98)	198 (57.55)
Careless	47 (12.02)	26 (55.31)
Ever tested for COVID-19		
No	185 (47.31)	93 (50.27)
Yes	206 (52.69)	131 (63.59)
Personal history of COVID-19 diagnosis		
No	341 (87.21)	191 (56.01)
Yes	50 (12.79)	33 (66)
Family member ever diagnosed with COVID-19		
No	226 (57.8)	125 (55.3)
Yes	165 (42.2)	99 (60)
Source of information regarding COVID-19 vaccine		
Ministry of Health newsletters/SMS	228 (58.31)	138 (60.52)
Social media	119 (30.43)	59 (49.57)
Newspaper and electronic media	15 (3.84)	10 (66.66)
Scientific publications/articles	17 (4.35)	12 (70.58)
Others	12 (3.07)	3 (25)

**Table 2:** Health and COVID-19 vaccine-related characteristics of participants (*n* = 391).

Health-Related Characteristics	Frequency *n* (%)	VA *n* (%)
Do you suffer from a chronic disease?		
No	345 (88.24)	197 (57.1)
Yes	46 (11.76)	27 (58.69)
Did you get the influenza vaccine during this influenza season?		
No	338 (86.45)	183 (54.14)
Yes	53 (13.55)	41 (77.35)
Do you intend to get the influenza vaccine for the next season?		
No	217 (55.5)	70 (32.25)
Yes	174 (44.5)	154 (88.5)
Would you get the seasonal influenza vaccine in the context of the COVID-19 pandemic?		
No	277 (70.84)	127 (45.84)
Yes	114 (29.16)	97 (85.08)
Do you have fears about COVID-19?		
No	183 (46.8)	95 (51.91)
Yes	208 (53.2)	129 (62.01)
Do you feel at risk of being infected by the new coronavirus?		
No	195 (49.87)	89 (45.64)
Yes	196 (50.13)	135 (68.87)
If a new vaccine for the COVID-19 virus were now available in the market, would you get vaccinated?		
No	167 (42.71)	167 (100)
Yes	224 (57.29)	224 (100)
Do you believe the new COVID-19 vaccine is safe, efficient and effective?		
No	139 (35.55)	24 (17.26)
Yes	252 (64.45)	200 (79.36)
Do you believe it is necessary to take the COVID-19 vaccine to protect yourself?		
No	137 (35.04)	10 (7.29)
Yes	254 (64.96)	214 (84.25)
Do you think that you will get COVID-19 in the future?		
No	258 (65.98)	128 (49.61)
Yes	133 (34.02)	96 (72.18)
We need to prioritise going back to our normal routines as soon as possible instead of worrying about protective behaviours.		
No	150 (38.6)	75 (50)
Yes	241 (61.64)	149 (61.82)
Are you willing be to volunteer for a clinical trial for a COVID-19 vaccine in Saudi Arabia?		
No	281 (71.87)	126 (44.83)
Yes	110 (28.13)	98 (89.09)
I think the actions of the Ministry of Health have helped reduce the spread of COVID-19.		
No	24 (6.14)	8 (33.33)
Yes	367 (93.86)	216 (58.85)
I trust the Ministry of Health to make good decisions about reducing the spread of COVID-19.		
No	17 (4.35)	4 (23.52)
Yes	374 (95.65)	220 (58.82)
Have you ever refused a vaccine for yourself or a child because you considered it useless or dangerous?		
No	336 (85.93)	197 (58.63)
Yes	55 (14.07)	27 (49.09)
Have you ever postponed a vaccine recommended by a physician?		
No	331 (84.65)	191 (57.7)
Yes	60 (15.35)	33 (55)
Have you ever had a vaccine for a child or yourself despite doubts about its efficacy?		
No	295 (75.45)	155 (52.54)
Yes	96 (24.55)	69 (71.87)

**Table 3:** Correlation results of vaccine acceptability and independent variables (*n* = 391).

Variable		Variable				
		Chronic Disease	Vaccine Hesitancy	Trust in the Health System	Perceived Fear or Risk	History of Previous Vaccination against Seasonal Influenza
Vaccine acceptability	Correlation coefficient (r)	0.002	0.001	0.126 *	0.133 **	0.557 **
	*p*-value	0.966	0.991	0.013	0.008	0.000

* Significance correlation at the 0.05 level (2-tailed), ** Significance correlation at the 0.01 level (2-tailed).

**Table 4:** Differences of vaccine acceptability based on location and monthly income.

(I) City of Residence		Mean Difference (I–J)	Std. Error	Sig.	95% Confidence Interval	
					Lower Bound	Upper Bound
Najran	Abha/Khamis Mushait	0.046	0.048	0.608	−0.067	0.158
	Jizan	−0.082	0.063	0.389	−0.230	0.065
Abha/Khamis Mushait	Najran	−0.046	0.048	0.608	−0.158	0.067
	Jizan	−0.128	0.061	0.094	−0.272	0.016
Jizan	Najran	0.082	0.063	0.389	−0.065	0.230
	Abha/Khamis Mushait	0.128	0.061	0.094	−0.016	0.272
(II) Monthly household income						
Less than 5000	5000 to 10,000	−0.048	0.060	0.705	−0.190	0.094
	More than 10,000	−0.111	0.057	0.123	−0.244	0.022
5000 to 10,000	Less than 5000	0.048	0.060	0.705	−0.094	0.190
	More than 10,000	−0.063	0.050	0.414	−0.180	0.054
More than 10,000	Less than 5000	0.111	0.057	0.123	−0.022	0.244
	5000 to 10,000	0.063	0.050	0.414	−0.054	0.180

**Table 5:** Path coefficients results through regression analysis.

Model			Unstandardized Coefficients			Std. Coefficients		*t*		Sig.	95.0% Confidence Interval for B	
			B		Std. Error	Beta					Lower Bound	Upper Bound
1	(Constant)		0.330		0.221			1.493		0.136	−0.105	0.765
	Vaccine Hesitancy		−0.101		0.064	−0.068		−1.582		0.114	−0.227	0.025
	Trust in Health System		0.231		0.102	0.097		2.255		0.025	0.030	0.432
	History of previous vaccination against seasonal influenza		0.666		0.056	0.515		11.890		0.000	0.556	0.776
	Perceived fear or risk		0.068		0.042	0.069		2.609		0.015	−0.015	0.150
a. Dependent Variable: Vaccine acceptability												
Model Summary (R-square value)												
Model		R		R Square			Adjusted R Square		Std. Error of the Estimate			
1		0.547 ^a^		0.299			0.292		0.362			

^a^. Predictors: (Constant), perceived fear or risk, vaccine hesitancy, trust in health system, history of previous vaccination against seasonal influenza.

## Data Availability

Data are available from the corresponding author for researchers who meet the criteria to access confidential data.

## Supplementary Materials

The following are available online at https://www.mdpi.com/article/10.3390/vaccines10010041/s1, Table S1: Group differences of male and female on vaccine acceptability; Table S2: Group differences between Saudi and non-Saudi for Vaccine acceptability; Table S3: Vaccine acceptability differences between medical and non-medical professionals; Table S4: Vaccine acceptability differences between careless and careful about health

## References

[1] Ratzan S.C. (2011). Vaccine literacy: A new shot for advancing health. J. Health Commun..

[2] Sallam M. (2021). COVID-19 vaccine hesitancy worldwide: A concise systematic review of vaccine acceptance rates. Vaccines.

[3] Dubé E., Gagnon D., Nickels E., Jeram S., Schuster M. (2014). Mapping vaccine hesitancy—Country-specific characteristics of a global phenomenon. Vaccine.

[4] MacDonald N.E., SAGE Working Group on Vaccine Hesitancy (2015). Vaccine hesitancy: Definition, scope and determinants. Vaccine.

[5] Kickbusch I.S. (2001). Health literacy: Addressing the health and education divide. Health Promot. Int..

[6] Sørensen K., Van den Broucke S., Fullam J., Doyle G., Pelikan J., Slonska Z., Brand H., (HLS-EU) Consortium Health Literacy Project European (2012). Health literacy and public health: A systematic review and integration of definitions and models. BMC Public Health.

[7] Chakraborty C., Sharma A.R., Sharma G., Bhattacharya M., Lee S.S. (2020). SARS-CoV-2 causing pneumonia-associated respiratory disorder (COVID-19): Diagnostic and proposed therapeutic options. Eur. Rev. Med. Pharmacol. Sci..

[8] Saha R.P., Sharma A.R., Singh M.K., Samanta S., Bhakta S., Mandal S., Bhattacharya M., Lee S.-S., Chakraborty C. (2020). Repurposing drugs, ongoing vaccine, and new therapeutic development initiatives against COVID-19. Front. Pharmacol..

[9] Chakraborty C., Lee S.-S., Sharma A., Bhattacharya M., Sharma G. (2020). The 2019 novel coronavirus disease (COVID-19) pandemic: A zoonotic prospective. Asian Pac. J. Trop. Med..

[10] Chakraborty C., Sharma A.R., Sharma G., Bhattacharya M., Saha R.P., Lee S.-S. (2020). Extensive partnership, collaboration, and teamwork is required to stop the COVID-19 outbreak. Arch. Med. Res..

[11] Barry M., Al Amri M., Memish Z.A. (2020). COVID-19 in the shadows of MERS-CoV in the kingdom of Saudi Arabia. J. Epidemiol. Glob. Health.

[12] Barry M., Ghonem L., Alsharidi A., Alanazi A., Alotaibi N., Al-Shahrani F., Majid F., BaHammam A. (2020). Coronavirus disease-2019 pandemic in the Kingdom of Saudi Arabia: Mitigation measures and hospital preparedness. J. Nat. Sci. Med..

[13] COVID-19 Vaccines: Development, Evaluation, Approval and Monitoring. https://www.ema.europa.eu/en/human-regulatory/overview/public-health-threats/coronavirus-disease-covid-19/treatments-vaccines/vaccines-covid-19/covid-19-vaccines-development-evaluation-approval-monitoring.

[14] CDC Developing COVID-19 Vaccines. https://www.cdc.gov/coronavirus/2019-ncov/vaccines/distributing/steps-ensure-safety.html.

[15] Setbon M., Raude J. (2010). Factors in vaccination intention against the pandemic influenza A/H1N1. Eur. J. Public Health.

[16] Gidengil C.A., Parker A.M., Zikmund-Fisher B.J. (2012). Trends in risk perceptions and vaccination intentions: A longitudinal study of the first year of the H1N1 pandemic. Am. J. Public Health.

[17] Larson H.J., Clarke R.M., Jarrett C., Eckersberger E., Levine Z., Schulz W.S., Paterson P. (2018). Measuring trust in vaccination: A systematic review. Hum. Vaccin. Immunother..

[18] Xiao X., Wong R.M. (2020). Vaccine hesitancy and perceived behavioral control: A meta-analysis. Vaccine.

[19] Reiter P.L., McRee A.-L., Gottlieb S.L., Brewer N.T. (2011). Correlates of receiving recommended adolescent vaccines among adolescent females in North Carolina. Hum. Vaccin..

[20] Covidvax.live. Live COVID-19 Vaccination Tracker-See Vaccinations in Real Time!. https://covidvax.live/location/sau.

[21] Al-Mohaithef M., Padhi B.K. (2020). Determinants of COVID-19 vaccine acceptance in Saudi Arabia: A web-based national survey. J. Multidiscip. Healthc..

[22] Quinn S.C., Jamison A.M., An J., Hancock G.R., Freimuth V.S. (2019). Measuring vaccine hesitancy, confidence, trust and flu vaccine uptake: Results of a national survey of White and African American adults. Vaccine.

[23] McRee A.-L., Brewer N.T., Reiter P.L., Gottlieb S.L., Smith J.S. (2010). The Carolina HPV immunization attitudes and beliefs scale (CHIAS): Scale development and associations with intentions to vaccinate. Sex. Transm. Dis..

[24] Reiter P.L., McRee A.-L., Pepper J.K., Gilkey M.B., Galbraith K.V., Brewer N.T. (2013). Longitudinal predictors of human papillomavirus vaccination among a national sample of adolescent males. Am. J. Public Health.

[25] Reiter P.L., McRee A.-L., Katz M.L., Paskett E.D. (2015). Human Papillomavirus vaccination among young adult gay and bisexual men in the United States. Am. J. Public Health.

[26] Killian M., Detoc M., Berthelot P., Charles R., Gagneux-Brunon A., Lucht F., Pulcini C., Barbois S., Botelho-Nevers E. (2016). Vaccine hesitancy among general practitioners: Evaluation and comparison of their immunisation practice for themselves, their patients and their children. Eur. J. Clin. Microbiol. Infect. Dis..

[27] Dubé E., Laberge C., Guay M., Bramadat P., Roy R., Bettinger J. (2013). Vaccine hesitancy: An overview: An overview. Hum. Vaccin. Immunother..

[28] Olive J.K., Hotez P.J., Damania A., Nolan M.S. (2018). The state of the antivaccine movement in the United States: A focused examination of nonmedical exemptions in states and counties. PLoS Med..

[29] Sarkar S., Zlojutro A., Khan K., Gardner L. (2019). Measles resurgence in the USA: How international travel compounds vaccine resistance. Lancet Infect. Dis..

[30] Burki T. (2020). The online anti-vaccine movement in the age of COVID-19. Lancet Digit. Health.

[31] Stoakes E. American Vaccine Misinformation and Extremism Are Infiltrating New Zealand. https://www.cnbc.com/2021/11/29/american-vaccine-misinformation-and-extremism-are-entering-new-zealand.html.

[32] Schraer R. Covid: Conspiracy and untruths drive Europe’s Covid protests. *BBC News*, 27 November 2021. https://www.bbc.com/news/59390968.

[33] Thousands Gather in Melbourne, Sydney, Gold Coast and across Australia in Orchestrated Protests against COVID-19 Vaccines, Restrictions. https://7news.com.au/lifestyle/health-wellbeing/thousands-gather-in-melbourne-sydney-gold-coast-and-across-australia-in-orchestrated-protests-against-covid-19-vaccines-restrictions-c-4724228.

[34] Haas E.J., Angulo F.J., McLaughlin J.M., Anis E., Singer S.R., Khan F., Brooks N., Smaja M., Mircus G., Pan K. (2021). Impact and effectiveness of mRNA BNT162b2 vaccine against SARS-CoV-2 infections and COVID-19 cases, hospitalisations, and deaths following a nationwide vaccination campaign in Israel: An observational study using national surveillance data. Lancet.

[35] Lopez Bernal J., Andrews N., Gower C., Robertson C., Stowe J., Tessier E., Simmons R., Cottrell S., Roberts R., O’Doherty M. (2021). Effectiveness of the Pfizer-BioNTech and Oxford-AstraZeneca vaccines on covid-19 related symptoms, hospital admissions, and mortality in older adults in England: Test negative case-control study. BMJ.

[36] Nordström P., Ballin M., Nordström A. (2021). Effectiveness of heterologous ChAdOx1 nCoV-19 and mRNA prime-boost vaccination against symptomatic COVID-19 infection in Sweden: A nationwide cohort study. Lancet Reg. Health-Eur..

[37] Thunstrom L., Ashworth M., Finnoff D., Newbold S. (2020). Hesitancy towards a COVID-19 vaccine and prospects for herd immunity. SSRN Electron. J..

[38] Fu C., Wei Z., Pei S., Li S., Sun X., Liu P. (2020). Acceptance and preference for COVID-19 vaccination in health-care workers (HCWs). MedRxiv.

[39] Harapan H., Wagner A.L., Yufika A., Winardi W., Anwar S., Gan A.K., Setiawan A.M., Rajamoorthy Y., Sofyan H., Mudatsir M. (2020). Acceptance of a COVID-19 vaccine in southeast Asia: A cross-sectional study in Indonesia. Front. Public Health.

[40] Gagneux-Brunon A., Detoc M., Bruel S., Tardy B., Rozaire O., Frappe P., Botelho-Nevers E. (2021). Intention to get vaccinations against COVID-19 in French healthcare workers during the first pandemic wave: A cross-sectional survey. J. Hosp. Infect..

[41] Wang K., Wong E.L.Y., Ho K.F., Cheung A.W.L., Chan E.Y.Y., Yeoh E.K., Wong S.Y.S. (2020). Intention of nurses to accept coronavirus disease 2019 vaccination and change of intention to accept seasonal influenza vaccination during the coronavirus disease 2019 pandemic: A cross-sectional survey. Vaccine.

[42] Lorenc T., Marshall D., Wright K., Sutcliffe K., Sowden A. (2017). Seasonal influenza vaccination of healthcare workers: Systematic review of qualitative evidence. BMC Health Serv. Res..

[43] Musa D., Schulz R., Harris R., Silverman M., Thomas S.B. (2009). Trust in the health care system and the use of preventive health services by older black and white adults. Am. J. Public Health.

[44] Harris K.M., Maurer J., Kellermann A.L. (2010). Influenza vaccine—Safe, effective, and mistrusted. N. Engl. J. Med..

[45] Lee C., Whetten K., Omer S., Pan W., Salmon D. (2016). Hurdles to herd immunity: Distrust of government and vaccine refusal in the US, 2002–2003. Vaccine.

[46] Al-awsat A. Middle-East Arab News Opinion. https://english.aawsat.com/home/article/3046376/who-praises-saudi-arabia%E2%80%99s-%E2%80%98impressive%E2%80%99-response-covid-19-pandemic.

