# Supplementary material for: Acceptability of the COVID-19 Vaccine among Adults in Saudi Arabia: A Cross-Sectional Study of the General Population in the Southern Region of Saudi Arabia
Source: Vaccines (Basel). 2021 Dec 29;10(1):41. doi: 10.3390/vaccines10010041 (PMC8777608; doi:10.3390/vaccines10010041)
Supplement: Supplementary file 1 [file vaccines-10-00041-s001.zip › vaccines-1460892-supplementary.pdf]

## T-test results of group differences on vaccine acceptability

**Table S1.** Group differences of male and female on vaccine acceptability.

| Gender | | | | | N | Mean | Standard Deviation | | Standard Error Mean | |
|---|---|---|---|---|---|---|---|---|---|---|
| Vaccine acceptability | Male | | | | 270 | 1.630 | 0.430 | | 0.026 | |
| | Female | | | | 121 | 1.606 | 0.432 | | 0.039 | |
| | Levene's Test for Equality of Variances | | t-test for Equality of Means | | | | | | | |
| | | F | Sig. | t | df | Sig. (2-tailed) | Mean Difference | Standard Error Difference | 95% CI Lower | 95% CI Upper |
| Vaccine acceptability | Equal variances assumed | 0.168 | 0.682 | 0.501 | 389.000 | 0.617 | 0.024 | 0.047 | -0.069 | 0.116 |
| | Equal variances not assumed | | | 0.499 | 229.535 | 0.618 | 0.024 | 0.047 | -0.069 | 0.117 |

**Table S2.** Group differences between Saudi and non-Saudi for Vaccine acceptability.

| Nationality | | | | | N | Mean | Standard Deviation | | Standard Error Mean | |
|---|---|---|---|---|---|---|---|---|---|---|
| Vaccine acceptability | Saudi | | | | 375 | 1.62 | 0.42 | | 0.02 | |
| | Non-Saudi | | | | 16 | 1.60 | 0.47 | | 0.11 | |
| | Levene's Test for Equality of Variances | | t-test for Equality of Means | | | | | | | |
| | | F | Sig. | t | df | Sig. (2-tailed) | Mean Difference | Standard Error Difference | 95% CI Lower | 95% CI Upper |
| Vaccine acceptability | Equal variances assumed | 1.52 | 0.21 | 0.17 | 389 | 0.86 | 0.01 | 0.10 | -0.19 | 0.23 |
| | Equal variances not assumed | | | 0.15 | 16.0 | 0.87 | 0.01 | 0.12 | -0.23 | 0.27 |

**Table S3.** Vaccine acceptability differences between medical and non-medical professionals.

| Profession | | | | | N | Mean | Standard Deviation | | Standard Error Mean | |
|---|---|---|---|---|---|---|---|---|---|---|
| Vaccine acceptability | Medical | | | | 141 | 1.62 | 0.43 | | 0.03 | |
| | Non-medical | | | | 250 | 1.62 | 0.42 | | 0.02 | |
| | Levene's Test for Equality of Variances | | t-test for Equality of Means | | | | | | | |
| | | F | Sig. | t | df | Sig. (2-tailed) | Mean Difference | Standard Error Difference | 95% CI Lower | 95% CI Upper |
| Vaccine acceptability | Equal variances assumed | 0.19 | 0.66 | 0.06 | 389 | 0.95 | 0.003 | 0.04 | -0.08 | 0.09 |

| | | | | | | | |
|---|---|---|---|---|---|---|---|
| Equal variances not assumed | 0.06 | 287.1 | 0.95 | 0.003 | 0.04 | -0.08 | 0.09 |

**Table S4.** Vaccine acceptability differences between careless and careful about health.

| Degree of carefulness about health | | N | Mean | Standard Deviation | Standard Error Mean |
|---|---|---|---|---|---|
| **Vaccine acceptability** | Careful | 344 | 1.63 | 0.42 | 0.02 |
| | Careless | 47 | 1.56 | 0.43 | 0.06 |

| | | Levene's Test for Equality of Variances | | t-test for Equality of Means | | | | | | |
|---|---|---|---|---|---|---|---|---|---|---|
| | | | | | | | | | 95% CI | |
| | | **F** | **Sig.** | **t** | **df** | **Sig. (2-tailed)** | **Mean Difference** | **Standard Error Difference** | **Lower** | **Upper** |
| **Vaccine acceptability** | Equal variances assumed | 0.43 | 0.51 | 0.93 | 389.0 | 0.35 | 0.06 | 0.06 | -0.06 | 0.19 |
| | Equal variances not assumed | | | 0.91 | 58.63 | 0.36 | 0.06 | 0.06 | -0.07 | 0.19 |
